# Efficacy and Tolerability of a Novel Topical Hydrator Used After Nonablative Laser Skin Rejuvenation

**DOI:** 10.1111/jocd.70968

**Published:** 2026-06-07

**Authors:** Sabrina Fabi, Priscilla Huang, Olivia Supan, Tsing Cheng, Elizabeth T. Makino

**Affiliations:** ^1^ Department of Dermatology University of California, San Diego San Diego California USA; ^2^ Allergan Aesthetics, an AbbVie company Irvine California USA

**Keywords:** combined modality therapy, dermal administration, hyaluronic acid, skin aging, skin care

## Abstract

**Background:**

Topical hyaluronic acid (HA) has shown promise for improving skin appearance, used alone or in combination with other facial rejuvenation procedures.

**Aims:**

To assess the efficacy and tolerability of a next‐generation topical hyaluronic acid–containing facial hydrator (HCH) when applied twice daily for 8 weeks after nonablative laser treatment.

**Methods:**

This 8‐week, single‐center pilot study was conducted among 10 healthy female (*n* = 9) and male (*n* = 1) participants (aged 28–65 years), with Fitzpatrick skin types II/III/IV, moderate to severe lack of dewiness, and global fine lines/wrinkles, who planned to undergo pre‐elective laser treatment. HCH was applied after laser treatment, then twice daily for 8 weeks. Investigator grading of skin quality and standardized photography were performed before and after laser treatment, immediately after initial HCH application, and at follow‐up visits (day 2; weeks 2/4/8); participant self‐assessments were conducted immediately after initial HCH application and at all follow‐up visits.

**Results:**

Following the first post‐laser HCH application, investigator assessments indicated significant clinical improvements in skin quality (less dryness/scaling, improved radiance) compared with assessments post‐laser/before HCH application. Continued improvement was noted in investigator‐graded skin quality throughout the 8‐week study. All participants expressed overall satisfaction with HCH. For the vast majority of questionnaire items pertaining to specific benefits of HCH, 90 to 100% of participants agreed/strongly agreed.

**Conclusions:**

The results of this 8‐week pilot study suggest that the novel skin hydrator HCH pairs well with an in‐office laser procedure, provides immediate and long‐term significant improvements in skin appearance and quality, and enhances the laser treatment experience.

## 1 Introduction

Skin appearance is considered an essential aspect of physical beauty. Healthy skin, often described as skin that is smooth, even toned, firm, dewy, and glowing, gives a youthful appearance relative to chronological age and is highly desired by individuals seeking to improve their appearance [1, 2]. Hydration is a key feature of healthy, radiant, and dewy‐looking skin, affecting not just visible attributes, but topographical (e.g., roughness, dryness, lines, pores) and mechanical (e.g., elasticity, pliability, firmness) qualities [1, 3, 4].

The skin contains natural humectants that regulate hydration by attracting and binding water [5]. These include urea, lactate, amino acids, and hyaluronic acid (HA), which is the primary regulator of skin hydration [5, 6]. HA is present in all body tissues and fluids but is particularly abundant in the skin; in fact, HA in the skin accounts for 50% of the body's total HA content [6]. HA helps to hydrate the dermis and epidermis [4] and its presence is critical for the hydration process. As a hygroscopic molecule, HA has exceptional water absorption capacity [4].

Skin aging is characterized by the disappearance of HA and other natural humectants, resulting in loss of skin moisture and ultimately contributing to skin dryness, roughness, dullness/lack of radiance, and the appearance of wrinkles [4, 5, 6, 7]. As a means of combatting this natural process, the use of topical humectants such as HA in moisturizers and hydrators can help draw water into the outer layers of the skin (stratum corneum) for an immediate but short‐term effect [8, 9]. Hydrators have been developed to additionally support and restore the natural levels of endogenous humectants (including HA), resulting in skin that is naturally more hydrated, thereby providing long‐term skin hydration and skin quality benefits [10]. The use of hydrators as a topical treatment has shown promise for improving skin quality, either on its own or in combination with other facial rejuvenation procedures, including fillers, microneedling, chemical peels, neurotoxins, and lasers [10, 11, 12, 13].

The next‐generation topical HA‐containing facial hydrator HCH was designed to improve skin hydration, both immediately and in the long‐term. Immediate effects are facilitated by the action of 5 different forms of HA: a time‐release HA designed to provide extended delivery, cross‐linked HA to provide lasting hydration, liposomally encapsulated HA for immediate hydration, non–cross‐linked HA for smoothness and hydration, and nano‐HA to soothe skin. The formula also has a collagen peptide component that has additional hydrating benefits. Additionally, HCH contains a novel blend of botanicals, including avocado oil esters, vitamin F, passion fruit seed oil, and extracts of the vitis flower stem and lotus sprout, which stimulate production of endogenous biohumectants in the skin, while also exerting beneficial effects on the dermal‐epidermal junction and epidermal skin barrier. HCH has been shown to increase endogenous levels of natural moisturizing factors and long‐chain ceramides in the stratum corneum and significantly improve skin quality parameters, including dewiness, hydrated appearance, smoothness, and radiance [14].

The use of carbon dioxide laser treatments as an ablative skin resurfacing technique has traditionally been the gold standard for treating skin issues such as acne scars and photoaging [15]. More recently, nonablative laser skin rejuvenation has become increasingly popular as a safer and less‐invasive modality for improving skin appearance [15, 16]. Nonablative laser treatments have been shown to improve the appearance of photoaged skin and reduce the appearance of pores, acne scars, wrinkles, and hyperpigmentation, contributing to a more youthful and radiant look [15, 16]. Side effects associated with nonablative skin rejuvenation are minimal [15, 16]; however, most patients experience redness and dryness during the recovery period [17]. Hydration is felt to play a critical role in the skin recovery process following laser treatment.

The aim of this pilot study was to assess the efficacy and tolerability of HCH when used over the course of 8 weeks after a single nonablative 1 440‐nm diode laser treatment.

## 2 Methods

### 2.1 Study Design and Participants

Healthy male and female participants aged ≥ 20 years with Fitzpatrick skin types I–VI who planned to receive a pre‐selected nonablative 1 440‐nm diode laser treatment (Clear & Brilliant; Solta Medical, Bothell, WA) were eligible to enroll in this 8‐week, single‐center exploratory study. Participants were identified and recruited from the study site's existing patient database. Participants were required to have moderate to severe fine lines/wrinkles (grade 4–9 on the Global Fine Lines/Wrinkles scale), moderate to severe lack of dewiness (grade 4–9 on the Dewy Hydrated Skin scale), and mild to severe overall photodamage on the face (grade 3–9 on the Overall Photodamage scale). Participants were required to agree to the following during the course of the study: avoid using any new products in the treatment area, including self‐tanners; maintain baseline eyelash and eyebrow condition; keep the treatment area free from facial hair that could interfere with study assessments; abstain from facial treatments that were not part of the study procedures (e.g., microdermabrasion, peels, facials, laser treatments, tightening treatments); and avoid direct and prolonged sun exposure as much as possible. Key exclusion criteria included any uncontrolled disease or preexisting dermatologic condition with the potential to interfere with study assessments; use of topical retinoids, skin‐lightening agents, or skin‐resurfacing treatments such as chemical peels, microdermabrasion, or microneedling within the previous 4 weeks; use of cosmetic injections or nonablative laser resurfacing other than the pre‐elected laser treatment within the previous 6 months; and use of oral retinoids or ablative procedures within the previous 12 months. Electrolysis, waxing, and depilatory use were not allowed on the treated area during the study.

The day of the laser treatment was designated as baseline (day 1). At this visit, participants were dispensed the study product (HCH; Allergan, an AbbVie company, Irvine, CA), and the first application to the full face was performed following the laser treatment. Participants were instructed to use HCH twice daily (morning and evening) along with a provided basic skincare regimen consisting of a facial cleanser (SkinMedica Facial Cleanser; Allergan, an AbbVie company), moisturizer (SkinMedica Ultra Sheer Moisturizer), and sunscreen (SkinMedica Essential Defense Mineral Shield 35 SPF Sunscreen; morning application only) for 8 weeks. Compliance with HCH was confirmed verbally throughout the study; in addition, new supplies were dispensed at each study visit, and used containers were collected.

### 2.2 Study Endpoints

Clinical investigator grading and standardized photography were performed pre–laser treatment, post–laser treatment, 15 min after HCH application post–laser treatment, and at each follow‐up visit (day 2 and weeks 2, 4, and 8). Participant self‐assessment questionnaires were conducted immediately after the initial HCH application and at each follow‐up visit. Except for the immediate post‐laser, post–initial HCH application assessments, all assessments involving skin evaluation were performed with clean skin and no topical products on the skin.

Clinical investigator assessments of efficacy consisted of grading participants' skin quality using separate, 10‐point clinical scales (0 = none; 1–3 = mild; 4–6 = moderate; 7–9 = severe) for the endpoints of dewy hydrated skin, global fine lines/wrinkles, redness/erythema, dryness/scaling, skin tone evenness, skin smoothness (visual), overall photodamage, and radiance (higher scores indicate poorer skin quality).

Standardized digital photographs were taken using a VISIA‐CR system (Canfield, Parsippany, NJ) to capture visual changes to the treatment area.

Participant self‐assessment questionnaires evaluating treatment effects and participants' experience using HCH were completed at baseline (immediately after application of HCH) and at each follow‐up visit. Participants were asked to assess any improvements immediately post‐application and with long‐term use of HCH, as well as their experience with the texture and application of the test product. They were also asked about their overall satisfaction and overall improvement in skin hydration and skin condition. Participants graded their level of agreement (agree strongly, agree, disagree, or disagree strongly) with statements regarding the look, feel, and texture of their skin and other treatment effects, and with statements regarding the texture and application experience of HCH. Level of satisfaction with the test product was graded as excellent (very satisfied), good (moderately satisfied), fair (slightly satisfied), or poor (not satisfied at all). The level of improvement in skin hydration and skin condition was graded as 0 (no change), 1 (slight improvement), 2 (moderate improvement), 3 (marked improvement), or 4 (complete clearing). Local cutaneous tolerability was assessed by participant grading of the degree of burning/stinging and itching on the full face, each evaluated separately using a 4‐point scale of 0 (none), 1 (mild), 2 (moderate), or 3 (severe). Adverse events were monitored and recorded throughout the study.

### 2.3 Statistical Analysis

Statistical analyses were conducted using the intent‐to‐treat (ITT) population, which included all participants who received treatment and participated in at least 1 postbaseline evaluation. As this was a small pilot study, sample size calculation was not performed. Percentage changes in investigator‐assessed skin quality endpoints from baseline to follow‐up timepoints were analyzed using the Wilcoxon signed‐rank test using the built‐in statistical functions of Excel. Results of participant self‐assessments were summarized descriptively. Statistical significance was defined as *p* < 0.05.

### 2.4 Ethical Considerations

The protocol and other study‐related materials were reviewed and approved by an independent institutional review board (Advarra IRB; registered with OHRP and FDA, IRB #00000971). The study was conducted in accordance with the Declaration of Helsinki and accepted standards for Good Clinical Practice. Participants provided informed consent prior to the initiation of any study‐related activity.

## 3 Results

### 3.1 Study Participants

The study enrolled 10 participants who met the eligibility criteria, including 9 females and 1 male (White, *n* = 8; mixed race, *n* = 2), all cisgender, with ages ranging from 28 to 65 years. The participants had Fitzpatrick skin types of II (*n* = 3), III (*n* = 6), and IV (*n* = 1). All 10 participants completed the 8‐week study.

### 3.2 Investigator Assessments

**FIGURE 1:**
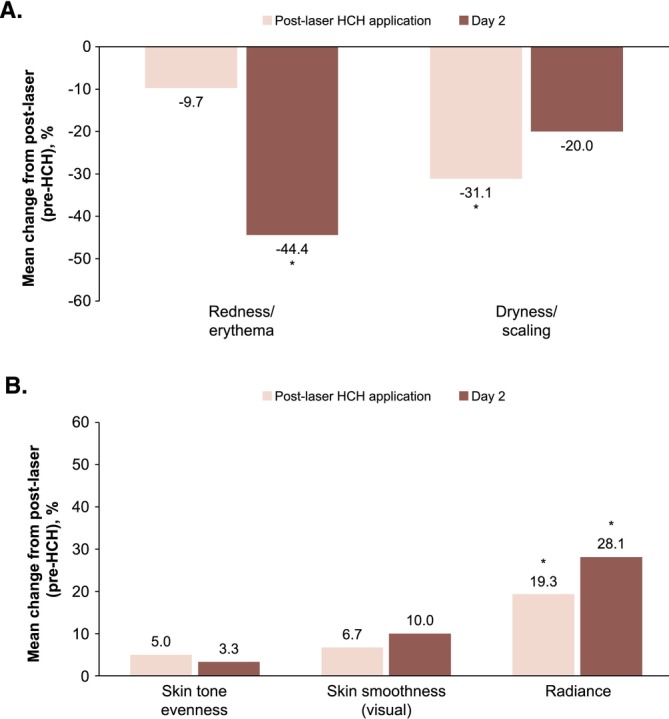
Mean percentage change from post‐laser to post‐application of HCH after laser and from post‐laser to day 2 in investigator‐assessed grading parameters. (A) Redness/erythema and dryness/scaling. (B) Skin tone evenness, skin smoothness (visual), and radiance. Overall photodamage results were not reported because changes were not expected by day 2. *p < 0.05.

When applied after laser treatment, investigator assessments indicated that HCH was associated with significant clinical improvements in participants' skin quality compared with post‐laser/pre‐HCH (Figure 1). Immediately after application of HCH, investigators noted a mean 31.1% decrease in dryness/scaling and a mean 19.3% increase in radiance (both *p* < 0.02 vs. post‐laser/pre‐HCH). Investigator assessments on day 2 indicated a 44.4% mean decrease in redness/erythema and a 28.1% mean increase in radiance as compared with post‐laser/pre‐HCH assessments (both *p* ≤ 0.004).

**FIGURE 2:**
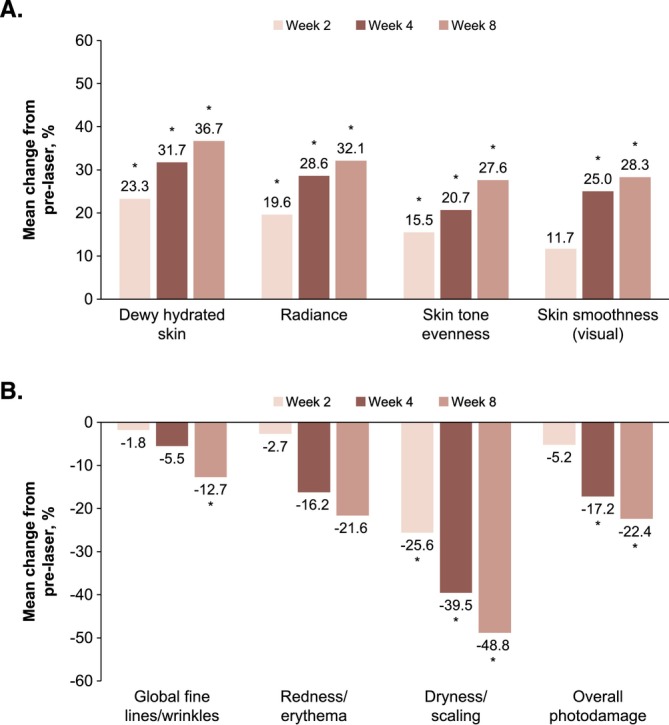
Mean percentage change from pre‐laser to weeks 2, 4, and 8 in investigator‐assessed grading parameters. (A) Dewy, hydrated skin, radiance, skin tone evenness, and skin smoothness (visual). (B) Global fine lines/wrinkles, redness/erythema, dryness/scaling, and overall photodamage. *p < 0.05.

Twice‐daily HCH application demonstrated long‐term improvements in participants' skin quality as assessed by investigators (Figure 2). As early as week 2, there were significant improvements from pre‐laser assessments in mean investigator grading scores for dewy hydrated skin (23.3%), radiance (19.6%), skin tone evenness (15.5%), and dryness/scaling (−25.6%) (all *p* ≤ 0.041). Grading scores for each of these assessments continued to show further improvement at weeks 4 and 8. The change in mean skin smoothness score from pre‐laser to week 2 improved (11.7%) at week 2 and achieved significance at week 4 (25.0%) and week 8 (28.3%) (both *p* ≤ 0.004). Similarly, improvements in overall photodamage were statistically significant at week 4 (−17.2%) and week 8 (−22.4%) (both *p* < 0.02). For global fine lines/wrinkles and redness/erythema, trends indicated ongoing improvements in mean scores at each visit, with global fine lines/wrinkles attaining statistical significance at week 8 (−12.7%; *p* = 0.031).

**FIGURE 3:**
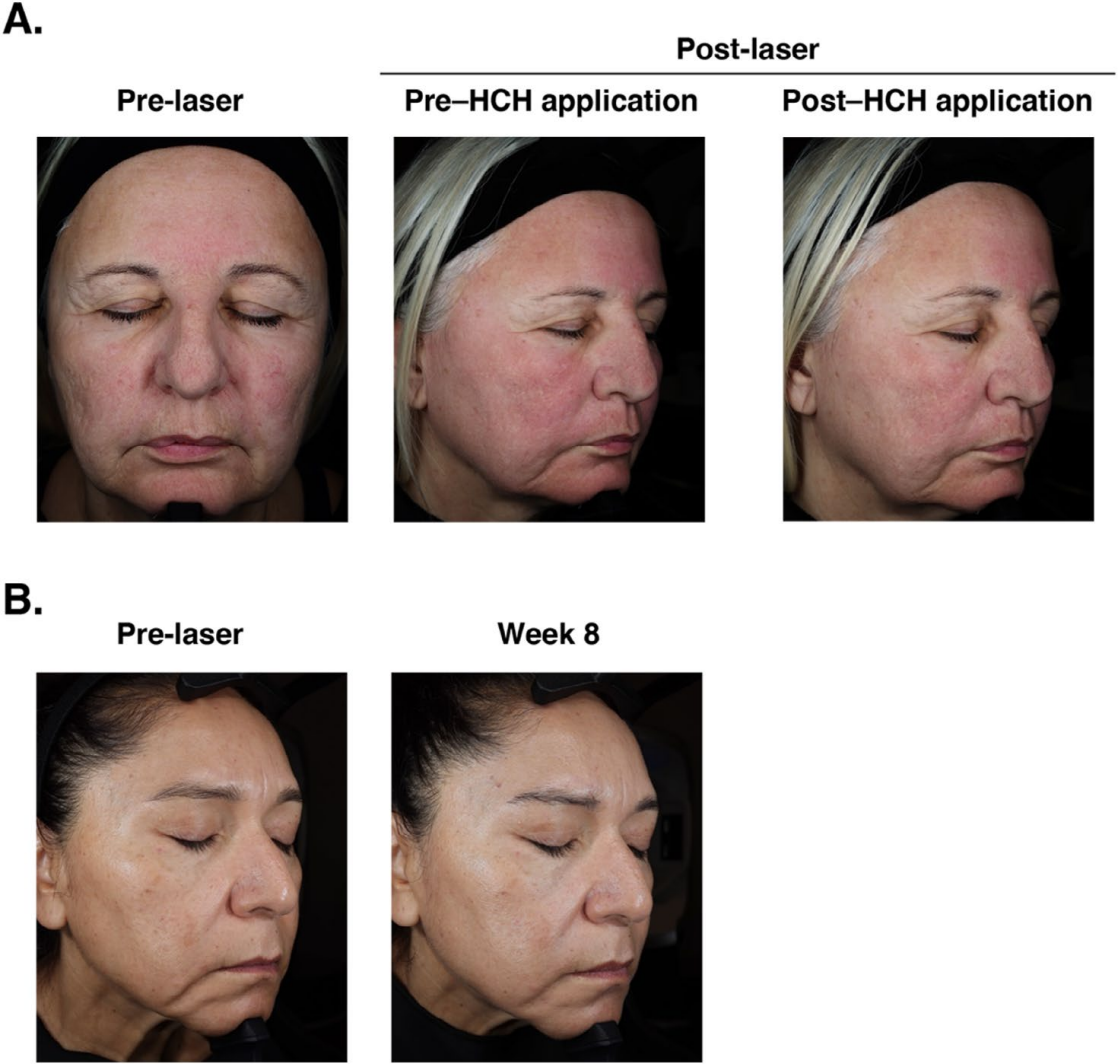
Representative participant photographs. (A) 65‐year‐old White female with Fitzpatrick skin type II pre‐laser, post‐laser, and immediately after application of HCH. (B) 54‐year‐old female of mixed race/ethnicity with Fitzpatrick skin type IV pre‐laser and at week 8 (no product on skin).

Representative participant photographs illustrating visible improvements in skin appearance from immediately following post‐laser to post‐application of HCH after laser and from pre‐laser to week 8 are presented in Figure 3.

### 3.3 Participant Assessments

**FIGURE 4:**
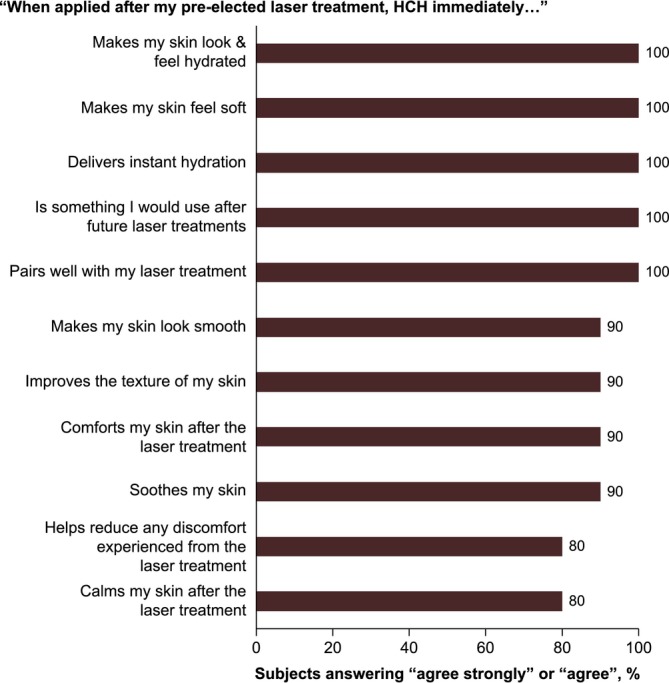
Participant self‐assessment responses at baseline immediately after application of HCH.

Participant self‐assessments performed immediately after application of HCH following the laser treatment indicated positive experiences with the treatment (Figure 4). All participants (100%) indicated that they agreed that HCH immediately made their skin look and feel hydrated, made their skin feel soft, delivered instant hydration, was something they would use after future laser treatments, and paired well with the laser treatment. Almost all participants (80% to 90%) agreed or strongly agreed with all other assessments.

**FIGURE 5:**
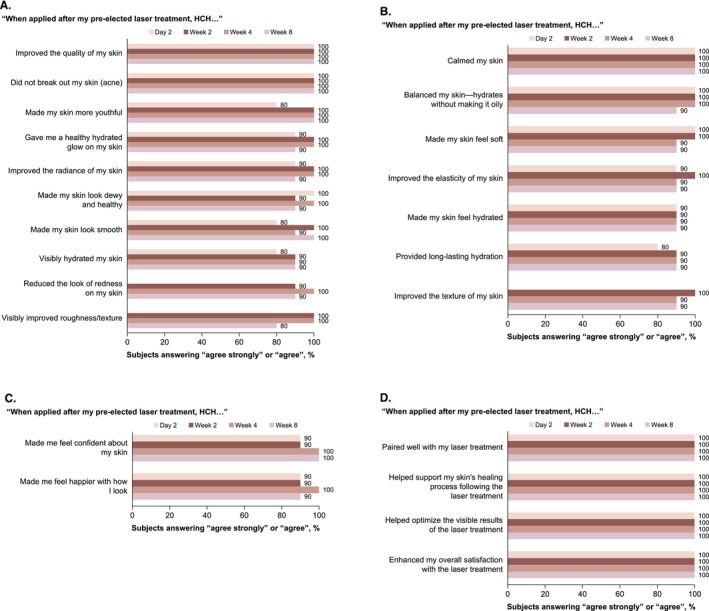
Participant self‐assessment responses at day 2 and weeks 2, 4, and 8. (A) Items related to skin appearance. (B) Items related to skin feel. (C) Items related to emotional impacts. (D) Items related to compatibility with laser treatment.

Participant satisfaction with the experience of using HCH after laser treatment was sustained throughout the 8‐week study period. At each follow‐up time point (day 2 and weeks 2, 4, and 8), 100% of participants agreed that HCH improved the quality of their skin, did not cause acne, and calmed their skin (Figure 5A,B). At each follow‐up time point, 80 to 100% of participants agreed with all statements regarding the beneficial effects of HCH on the appearance and feel of their skin as well as emotional impacts, including feeling more confident about their skin and feeling happier with how they look (Figure 5A–C). At all follow‐up timepoints, 100% of participants agreed that HCH paired well with the laser treatment, helped support their skin's healing process after the procedure, helped optimize the visible results of the laser treatment, and enhanced their overall satisfaction with the laser treatment (Figure 5D).

**FIGURE 6:**
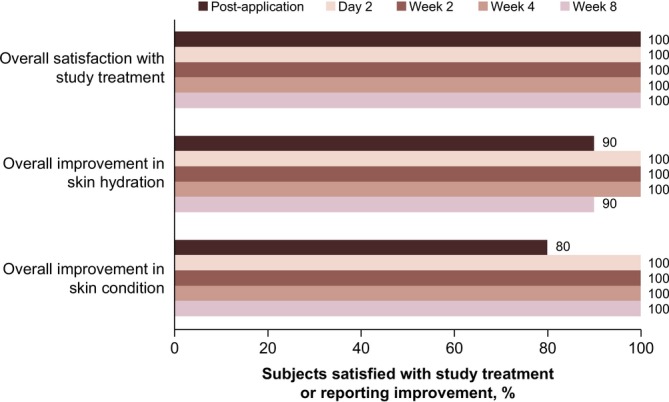
Participant overall satisfaction^a^ with HCH when combined with a laser treatment and self‐assessed overall improvement^b^ in skin hydration and condition immediately post‐application and at day 2 and weeks 2, 4, and 8. ^a^Responses of “Excellent (very satisfied),” “Good (moderately satisfied),” and “Fair (slightly satisfied)” were categorized as satisfaction. ^b^Responses of “Slight improvement,” “Moderate improvement,” “Marked improvement,” and “Complete clearing” were categorized as improvement.

**FIGURE 7:**
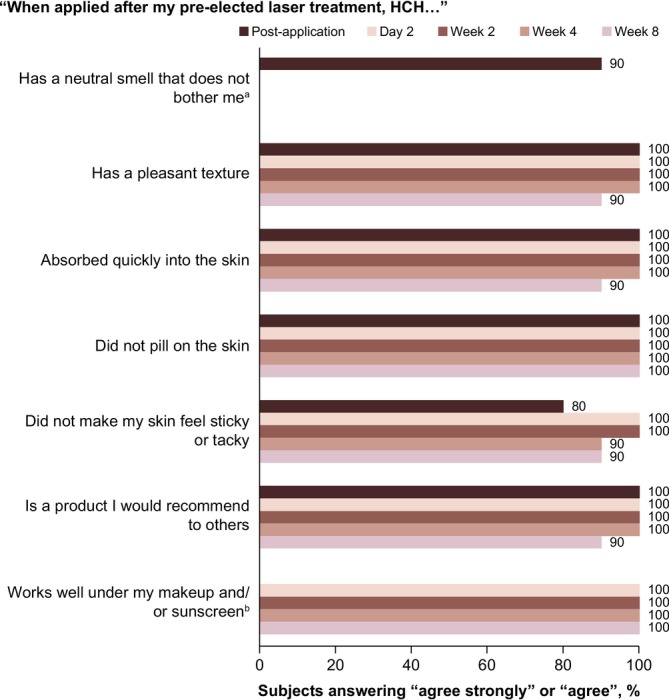
Participant questionnaire responses related to texture and application of HCH immediately post‐application and at day 2 and weeks 2, 4, and 8. ^a^Asked at post‐application only. ^b^Asked at follow‐up visits only.

After the first application and at all follow‐up time points, 100% of participants reported overall satisfaction with HCH; moreover, overall improvement in skin hydration and skin condition was reported by 90 to 100% and 80% to 100% of participants, respectively (Figure 6). Participants also reported positive feedback regarding the texture and application of HCH throughout the study period (Figure 7). Nearly all participants reported that HCH had a pleasant texture and worked well under their makeup and/or sunscreen.

### 3.4 Safety and Tolerability

HCH was well tolerated, with no HCH‐related adverse events reported. All participant‐assessed grades for itching remained at 0 (none) throughout the study. Additionally, upon application, HCH reduced burning/stinging from laser treatment from a mean score of 1.2 to 0.6 (50% decrease). By day 2, burning/stinging resolved (100% decrease) to a mean score of 0.

## 4 Discussion

This pilot study assessed the efficacy and tolerability of HCH, a next‐generation topical HA‐containing facial hydrator, when used after a nonablative laser treatment. Our findings suggest that HCH produced a rapid improvement in skin quality, reducing dryness, scaling, and redness while enhancing hydration and radiance, as reported by both investigators and participants. Benefits were evident immediately after application and continued to improve over 8 weeks of twice‐daily use. All parameters apart from redness/erythema were significantly improved by the end of study week 8; although, it is possible that redness/erythema may have achieved statistically significant improvement with a longer duration of follow‐up. HCH was also well tolerated.

Further, results from this study suggest that significant reductions in dryness/scaling and redness/erythema were observed immediately after application of HCH and on day 2. Anticipated downtime following an aesthetic procedure can be a major barrier to treatment, and the ability to return as quickly as possible to normal daily activities could positively influence patient satisfaction. Although side effects linked with nonablative laser treatment are minimal [15, 16], most patients do still experience redness and dryness during the recovery period [17]. Individuals seek aesthetic interventions that integrate seamlessly into their routines, and even short periods of social or occupational disruption may deter them from pursuing or repeating treatment. Consequently, products that help shorten recovery time, improve immediate post‐procedure appearance, or enhance overall comfort may support broader acceptance and adherence to aesthetic regimens. Interventions that deliver early improvements in skin quality, such as HCH, can offer meaningful clinical and quality‐of‐life benefits, reinforcing both patient confidence and satisfaction with the treatment experience.

Given the multitude of factors known to contribute to the aging process, a multimodal approach to improving skin quality may enhance results and improve patient satisfaction [2, 18]. A prior formulation of this product was shown in clinical studies to improve various features of skin quality when used alone [10], after injectable neuromodulator treatment [11], and after chemical (retinol‐ or acid‐based chemical peels), physical (microdermabrasion, microneedling, dermaplaning), or energy‐based (laser skin resurfacing, intense pulsed light photo‐rejuvenation, radiofrequency, ultrasound) aesthetic procedures [13]. The next‐generation formulation has double the amount of HA as compared with the original formulation and contains additional proprietary technology designed to help support and replenish the skin's natural HA and other humectant levels and thereby enhance its ability to attract and retain moisture for long‐term skin quality benefits.

For medical procedures and treatments with the primary goal of achieving aesthetic benefits, patient satisfaction is considered a major determinant of treatment “success.” [19] In this study, not only did satisfaction with HCH remain at 100% throughout the study period, but participant responses also suggested that HCH was an excellent companion to their nonablative laser treatment, supporting the healing of their skin, optimizing the outcomes of their nonablative laser procedure, and enhancing overall satisfaction with their skin rejuvenation procedure. This finding was not surprising, given the complementary nature of the types of skin improvements expected with each type of intervention. Nonablative laser treatment offers important skin benefits, such as addressing photodamaged areas and fostering improvements in skin tone, texture, fine lines, and dyschromia [16, 20]; HCH promotes distinctive and additive improvements, such as promoting a dewy, hydrated appearance and improving dryness/scaling and skin radiance.

Although this study shows promising results, it does have limitations. One limitation of this research is the small size of the study group and the lack of a control group. In addition, the endpoints were mainly subjective in nature; however, subjective patient perceptions of outcomes for this type of intervention are certainly a highly relevant aspect of any measure of benefit. Further, endpoint gradings made by investigators tracked similarly to those of participants. Given the exploratory design and limited sample size, these findings should be interpreted as preliminary, underscoring the need for a larger, randomized controlled trial to confirm the observed effects and support broader generalizability.

## 5 Conclusions

The daily use of the novel topical hydrator HCH after nonablative 1 440‐nm diode laser skin resurfacing enhanced patient outcomes and overall satisfaction with laser treatment. There were both immediate and long‐term clinical improvements noticeable by both clinicians and study participants. The data from this pilot study suggest potential benefits for patients seeking skin rejuvenation therapy, and also for clinical practices that are seeking to enhance nonablative laser therapy regimens. This multimodal approach to skin rejuvenation could contribute to maximizing patient satisfaction with such procedures.

## Author Contributions

All authors have read and approved the final manuscript. S.F. provided the collection and assembly of data; S.F., P.H., O.S., T.C., and E.T.M. provided data interpretation; S.F. provided the study design; and S.F., P.H., O.S., T.C., and E.T.M. reviewed the manuscript, made revisions, and gave final approval of the manuscript.

## Funding

Allergan Aesthetics, an AbbVie company, funded this study and participated in the study design, research, analysis, data collection, interpretation of data, review, and approval of the publication. All authors had access to relevant data and participated in the drafting, review, and approval of this publication. No honoraria or payments were made for authorship. Medical writing support was provided by Sandra Westra, PharmD of Peloton Advantage LLC (an OPEN Health company) and funded by Allergan Aesthetics, an AbbVie company.

## Disclosure

Sabrina Fabi is a clinical investigator, speaker, and consultant for AbbVie. Priscilla Huang, Tsing Cheng, Olivia Supan, and Elizabeth T. Makino are full‐time employees of AbbVie and may own AbbVie stock.

## Ethics Statement

The protocol and other study‐related materials were reviewed and approved by an independent institutional review board (Advarra IRB). The study was conducted in accordance with the Declaration of Helsinki and accepted standards for Good Clinical Practice. Participants provided informed consent prior to the initiation of any study‐related activity.

## Consent

All patients provided written consent for their photographs to be published.

## Conflicts of Interest

Sabrina Fabi is a clinical investigator, speaker, and consultant for AbbVie. Priscilla Huang, Tsing Cheng, Olivia Supan, and Elizabeth T. Makino are full‐time employees of AbbVie, and may own AbbVie stock.

## Data Availability

AbbVie is committed to responsible data sharing regarding the clinical trials we sponsor. This includes access to anonymized, individual and trial‐level data (analysis data sets), as well as other information (eg, protocols, clinical study reports, synopses, or statistical analysis plans), as long as the trials are not part of an ongoing or planned regulatory submission. This clinical trial data can be requested by any qualified researchers who engage in rigorous, independent scientific research, and will be provided following review and approval of a research proposal and Statistical Analysis Plan (SAP) and execution of a Data Use Agreement (DUA). Data requests can be submitted at any time after approval in the US and Europe and after acceptance of this manuscript for publication. The data will be accessible for 12 months, with possible extensions considered. For more information on the process, or to submit a request, visit the following link: https://vivli.org/ourmember/abbvie/ then select “Home”.
